# High prevalence of *bla*_CTX‐M‐15_ type extended‐spectrum beta‐lactamases in Gambian hooded vultures (*Necrosyrtes monachus*): A threatened species with substantial human interaction

**DOI:** 10.1002/mbo3.1349

**Published:** 2023-03-23

**Authors:** Hanna Woksepp, Fagimba Camara, Jonas Bonnedahl

**Affiliations:** ^1^ Department of Research Region Kalmar County Kalmar Sweden; ^2^ Department of Chemistry and Biomedical Sciences Linnaeus University Kalmar Sweden; ^3^ Department of Wildlife Management, Abuko The West African Birds Study, Association (WABSA) Serrekunda Gambia; ^4^ Department of Biomedical and Clinical Sciences Linköping University Linköping Sweden; ^5^ Department of Infectious Diseases Region Kalmar County Kalmar Sweden

**Keywords:** antibiotic resistance, environmental microbiology, infectious agents, microbial ecology, microbiological‐based health strategies

## Abstract

One hundred fecal samples from hooded vultures in the Gambia (Banjul area) were investigated for the presence of bacteria with extended‐spectrum cephalosporin‐ (ESBL/AmpC), carbapenemases, and colistin resistance. No Enterobacteriales carrying carbapenemases or resistance against colistin were detected. Fifty‐four ESBL‐producing *Escherichia coli* and five ESBL‐producing *Klebsiella pneumoniae* isolates were identified in 52 of the samples, of which 52 *E. coli* and 4 *K. pneumoniae* yielded passed sequencing results. Fifty of the *E. coli* had ESBL phenotype and genotype harboring *bla*_CTX‐M_ genes, of which 88.5% (*n* = 46) were the *bla*_CTX‐M‐15_ gene, commonly found on the African continent. Furthermore, the genetic context around *bla*_CTX‐M‐15_ was similar between isolates, being colocalized with IS*Kpn19*. In contrast, cgMLST analysis of the *E. coli* harboring ESBL genes revealed a genetic distribution over a large fraction of the currently known existing *E. coli* populations in the Gambia. Hooded vultures in the Gambia thus have a high ESBL *E. coli*‐prevalence (>50%) with low diversity regarding key resistance genes. Furthermore, given the urban presence and frequent interactions between hooded vultures and humans, data from this study implies hooded vultures as potential vectors contributing to the further dissemination of antibiotic‐resistance genes.

## Graphical abstract

In this study, we found that hooded vulture has a high prevalence of ESBL‐producing bacteria. Based on these data, hooded vultures as top predators may function as a bioindicator for the presence of antibiotic‐resistant bacteria (ARB). Furthermore, this study highlights how vultures are exposed to anthropogenic impact, not only posing a threat to the birds but also serving as vectors for further dissemination of ARB in the environment and transmission to humans.

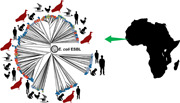

## 1 INTRODUCTION

Antimicrobial resistance (AMR) increases healthcare costs, aggravates the treatment of infectious diseases, and may severely impede progress in public health such as childhood survival and agriculture (Cosgrove, 2006; Holmes et al., 2016; Laxminarayan et al., 2016). Today, studies stress the importance of community‐based transmission of antimicrobial‐resistant bacteria (ARB) and hospital‐acquired infections (Onduru et al., 2021). It has also been recognized that the transmission of ARB between animals and humans may have a larger impact than previously thought (Dolejska & Literak, 2019; Laxminarayan et al., 2020; Swift et al., 2019). Furthermore, the presence of ARB in the environment could also pose a risk for horizontal gene transfer from resistant to nonresistant bacteria, increasing ARB reservoirs and enable new transmission routes (Bengtsson‐Palme et al., 2019; Guo et al., 2017). Extended‐spectrum beta‐lactamases (ESBL)‐producing and carbapenemases‐producing Enterobacteriales are classified as a major threat from a One Health perspective (Laxminarayan et al., 2016; Manenzhe et al., 2015; Onduru et al., 2021). Africa has been identified as the continent with the highest number of circulating antibiotic‐resistant genes (ARG) (Sekyere & Reta, 2020), where *bla*_CTX‐M‐15_ dominates among the ESBL‐producing bacteria (Bachiri et al., 2017; Fortini et al., 2015; Mshana et al., 2013; Onduru et al., 2021). Other ARGs often identified among ARB in Africa include *bla*_TEM‐1_, *qnrA/B/D/S*, *sul1/2/3*, *mcr‐1*, and *catA/B* (Sekyere & Reta, 2020). The prevalence of carbapenemase‐producing bacteria in Africa is still inadequately investigated, but there is an increase in the detection, especially of class B and D carbapenemases such as *bla*_IMP_, *bla*_VIM,_ and *bla*_OXA‐48_ (Manenzhe et al., 2015; Sekyere & Reta, 2020).

The hooded vulture (*Necrosyrtes monachus*) is a small (~70 cm and ~2.1 kg) species of vulture, mostly brown, with a wingspan up to 180 cm (BirdLife International, 2022). The species has been listed as critically endangered since 2015 by Birdlife International (BirdLife International, 2021, 2022). Major concerns include intentional and secondary poisoning, hunting, both persecution and unintentional, and loss of habitats (BirdLife International, 2021, 2022; Ogada et al., 2012). There have also been reports of vulnerability to avian influenza (BirdLife International, 2021, 2022). Furthermore, vultures are sensitive to diclofenac, and even low levels cause kidney failure and death (Ogada et al., 2012). In the West African region, the decline rate of vultures is high (Henriques et al., 2018), although the coastal zone of Gambia appears to have the largest population estimated at 7000–10,500 in a 600 km^2^ sampled area, where it is the most common vulture species in the area (BirdLife International, 2021, 2022; Mawdo Jallow et al., 2016). Hooded vultures are at the top of the food web and feed mainly on carrions and insects (BirdLife International, 2021; Henriques et al., 2018). Vultures provide ecosystem services as scavengers contributing to nutrient cycling by consuming organic waste and by competing and thereby controlling other scavengers (Henriques et al., 2018). Thus, vultures may be bioindicators for contaminants, biocides, and other anthropogenic pollution. Recent reports suggest that over 20% of different vulture species in both Europe (Canary Islands) and Asia carries cefotaxime‐resistant *Escherichia coli* isolates (Carvalho et al., 2020). In this study, the prevalence of resistant *E. coli* and *Klebsiella pneumoniae* was investigated in fecal samples from hooded vultures in the Gambia.

## 2 MATERIALS AND METHODS

### 2.1 Sampling

One hundred fecal samples from hooded vultures (*N. monachus*) were collected on 13–14th November 2019 by swabbing freshly deposited feces. Samples were collected by placing plastic sheets under two different trees used for nightly roost by a large number of hooded vultures. The roosting trees were located in the greater Banjul area, separated by 10 km. The number of samples collected was less than the number of birds roosting in a respective tree. The Copan swabs were stored in Amies transport medium at <8°C. Within 1 week of sampling, the samples were shipped to Sweden.

### 2.2 Bacterial culturing

**Figure 1:**
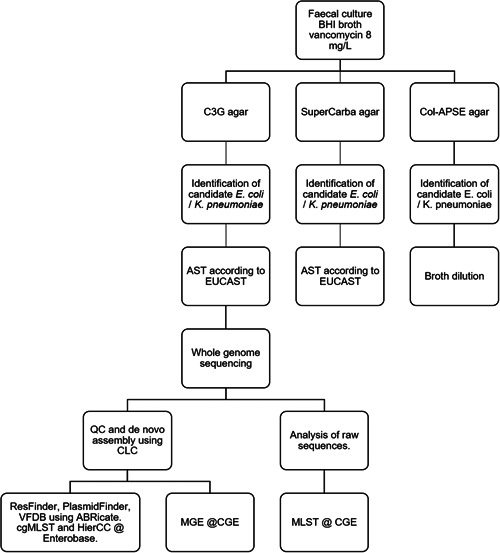
Workflow and sample handling.

At arrival on 22nd Nov 2019, the samples were inoculated in 2 mL brain heart infusion (BHI) broth (Becton Dickinson) with 8 mg/L vancomycin (Sigma‐Aldrich), and the broth was incubated for 18–24 h at 36°C in aerobic conditions. For selective screening of cephalosporin‐resistant bacteria, 10 µL overnight BHI broth was inoculated on CHROMagar™ C3G^R^ agar (CHROMagar™, France). For selective screening of carbapenem‐resistant bacteria, 10 µL overnight BHI broth was inoculated on mSuperCarba™ agar (CHROMagar™, France). To selectively screen putative colistin‐resistant bacteria, 10 µL overnight BHI broth was inoculated on Col‐APSE agar (CHROMagar™, France). All plates were incubated for 18–24 h at 36°C in aerobic conditions; see Figure 1 for analysis workflow. *E. coli* CCUG 17620 was used as a negative control for C3G^R^, mSuperCarba™ and Col‐APSE agar plates. *K. pneumoniae* CCUG 45421 and CCUG 64452 were used as a positive control for C3G^R^ and mSuperCarba™ agar plates, respectively. *E. coli* CCUG 70662 was used as a positive control for Col‐APSE agar plates.

### 2.3 Isolation and identification

Putative *E. coli* and *K. pneumoniae* colonies were isolated from C3G^R^, mSuperCarba™ and Col‐APSE agar. In most cases, one putative colony per plate was selected, although depending on colony morphology, more than one was chosen in some cases. Identification was performed using matrix‐assisted laser desorption ionization time‐of‐flight (MALDI TOF) mass spectrometry (Bruker Daltonics) according to the protocol from the manufacturer. Threshold >2 and database versions BDAL 5989 and 6903 were used for bacterial identification (Seng et al., 2009). Antimicrobial susceptibility testing, according to the European Committee on Antimicrobial Susceptibility Testing (EUCAST), was performed for identified *E. coli* and *K. pneumoniae* with antibiotic discs for ampicillin (10 µg), cefadroxil (30 µg), chloramphenicol (30 µg), ciprofloxacin (5 µg), gentamicin (10 µg), mecillinam (10 µg), nalidixic acid (30 µg), nitrofurantoin (100 µg), meropenem (10 µg), piperacillin‐tazobactam (30 + 6 µg), tetracycline (30 µg), trimethoprim (5 µg), and trimethoprim‐sulfamethoxazole (1.25 + 23.75 µg) (Thermo Fisher Scientific Oxoid Ltd) (EUCAST, 2021). The selection of antibiotics for AST was based on antibiotics used for screening for any resistance mechanism by the Swedish national veterinary institute. Interpretation of inhibition zone diameters was done according to EUCAST breakpoints (EUCAST, 2021), except for tetracycline, where the Normalized Resistance Interpretation method was used (Kronvall & Smith, 2016). Isolates resistant to ampicillin and cefadroxil were also tested for ESBL phenotype using a double‐disk synergy test (DDT) with amoxicillin‐clavulanic acid (31 µg), cefepime (30 µg), cefotaxime (30 µg), cefoxitin (10 µg), and ceftazidime (30 µg) (Thermo Fisher Scientific Oxoid Ltd). Broth microdilution (Micronaut‐S, Merlin Diagnostika) was used for AST against colistin for isolates collected from Col‐APSE agar.

### 2.4 Genomic analysis

DNA was extracted from all *E. coli* (*n* = 54) and *K. pneumoniae* (*n* = 5) colonies isolated from C3G agar using MagNA Pure Compact total nucleic acid isolation kit (Roche). Preparation of multiplexed DNA libraries was done using NexteraXT or Nextera DNA Flex library preparation kits (Illumina). The whole genome sequencing was performed using HiSeq. 4000 (Illumina).

Raw reads were trimmed and filtered using CLC genomic workbench version 21.0.4 (Qiagen) with default settings. De novo assembly and QC were performed using CLC genomic workbench version 21.0.4 (Qiagen) with default settings. In silico analysis of multilocus sequence type (MLST) (Larsen et al., 2012) was done by uploading raw fastq.gz sequences to the Center for Genomic Epidemiology. De novo assembled sequences were analyzed using ABRicate (Seeman, https://github.com/tseemann/abricate) for the detection of antibiotic resistance genes (Bortolaia et al., 2020; Clausen et al., 2018; Zankari et al. 2017), virulence genes (Chen et al., 2016) and detection of plasmid replicons (Carattoli et al., 2014; Clausen et al., 2018). The prevalence of mobile genetics elements (Johansson et al., 2021) and phylogroup typing (Beghain et al., 2018) was assessed by uploading preassembled fasta files to the Center for Genomic Epidemiology and ClermonTyping (http://clermontyping.iame-research.center/), respectively. The sequences from the *E. coli* isolates in this study were compared to all other existing *E. coli* isolates in Enterobase reported to be collected in Gambia (*n* = 723) using hierarchical clustering of cgMLST data with the cgMLST V1 + HierCC V1 scheme with NINJA NJ algorithm (Zhou et al., 2018, 2020).

## 3 RESULTS AND DISCUSSION

### 3.1 Phenotypic and genotypic resistance characterization

**Figure 2:**
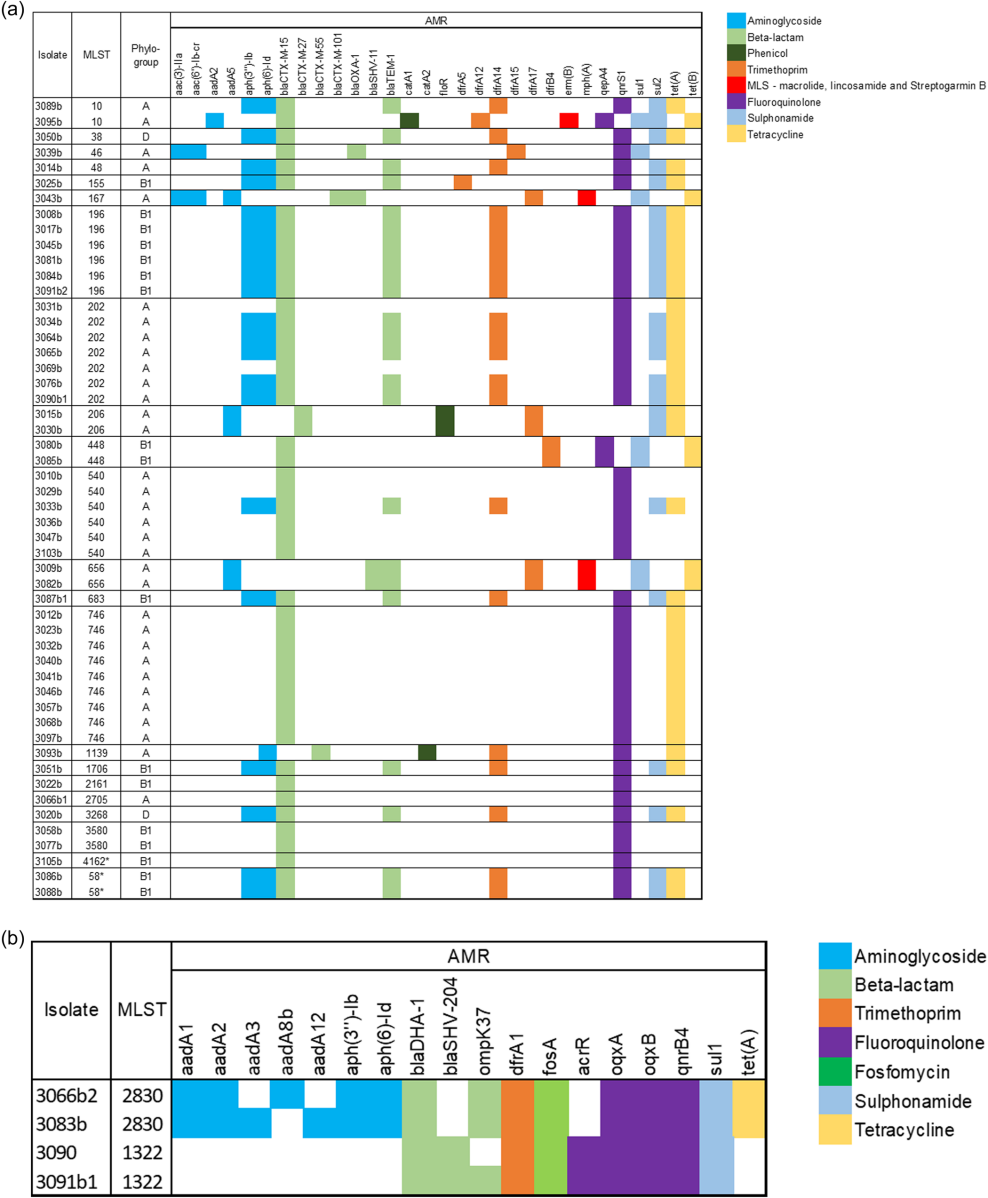
The genomic presence of antimicrobial resistance genes indicated by colored squares, grouped in antimicrobial classes according to color for each ESBL *Escherichia coli* isolate (a) and *Klebsiella pneumoniae* isolate (b). Isolates are grouped according to multilocus sequence type (MLST) results.

Screening for antibiotic‐resistant *E. coli* and *K. pneumoniae* from 100 fecal samples from hooded vultures was performed using selective growth medium for extended‐spectrum cephalosporin‐ (ESBL/AmpC), carbapenem‐ and colistin resistance (i.e., C3G, SuperCarba and Col‐APSE agar). In the selective screen for extended‐spectrum cephalosporin resistance (C3G agar) *E. coli* were isolated from 54/100 samples, for which sequencing was successful in 52, whereas *K. pneumoniae* were isolated in 5/100 samples for which sequencing was successful in four. Out of the 52 sequenced *E. coli*, ESBL *bla*_CTX‐M_‐genotype was identified in 50 and *bla*_SHV_ in two (Figure 2a), whereas none of the four sequenced *K. pneumoniae* showed an ESBL genotype. All *E. coli* with ESBL‐genotype had ESBL phenotype being resistant to 3rd and/or 4th generation extended‐spectrum cephalosporins with a positive synergy test when combined with clavulanic acid (Authority et al., 2020). Isolates with unsuccessful sequencing were excluded from further analysis. The *K. pneumoniae* isolates were all resistant to cefadroxil, ciprofloxacin, nalidixic acid, trimethoprim, and trimethoprim‐sulfamethoxazole (Supporting Information: Table S1 at https://doi.org/10.5281/zenodo.7645125) and had resistance genes *bla*_DHA‐1_, *dfrA1*, *fosA*, *oqxA*, *oqxB*, *qnrB4*, and *sul* (Figure 2b). *K. pneumoniae* lacks chromosomal‐inducible AmpC enzymes (Hennequin et al., 2018), and thus *bla*_DHA_ was probably located on a plasmid, although it could not be confirmed due to too short contigs around the gene.

No *E. coli* or *K. pneumoniae* were isolated from SuperCarba agar. Seven isolates, four *E. coli*, and three *K. pneumoniae* were isolated from Col‐APSE agar, but none had phenotypic resistance against colistin when assessed using broth microdilution, and no further analysis was done.

All of the *E. coli* isolates and the *K. pneumoniae* isolates from the selective screening for extended‐spectrum cephalosporin resistance (*n* = 52 for *E. coli*, *n* = 4 for *K. pneumoniae*, where three samples contained both *E. coli* and *K. pneumoniae*) were defined as multidrug‐resistant (MDR) having resistance against ≥1 agent in ≥3 different antimicrobial categories (Supporting Information: Table S1 at https://doi.org/10.5281/zenodo.7645125) (Magiorakos et al., 2012). Thus at least 53% of the vultures carried MDR bacteria, which is higher than in a recent study with a comparable methodology that found 40% MDR *E. coli* in cloacal samples derived from Egyptian vultures in the Canary Islands (Suarez‐Perez et al., 2021). Furthermore, the prevalence of ESBL in this study (>50%) is higher than reported from vultures in the Canary Islands, where 22.7% (*n* = 5/22) had ESBL phenotype (Carvalho et al., 2020). Blanco et al. also reported lower frequencies (25%–30%) in both griffon and Egyptian vultures breeding in Spain (Blanco et al., 2020). In comparison, at the time of the study, the prevalence of *E. coli* resistant to 3rd generation cephalosporins in Spain was 10%–25% (ECDC, 2022). The frequency of ESBL‐producing *E. coli* in Andean condors in Chile in 2019 was 63% (*n* = 17/27), and the dominating genes were *bla*_CTX‐M‐14_ and *bla*_CTX‐M‐55_ (Fuentes‐Castillo et al., 2020). Hernandez et al. showed high frequencies of ESBL‐producing *E. coli* (30%) among avian samples already in 2009 (Hernandez et al., 2013). White‐tailed eagles from the Nature Reserve Gornje Podunavlje with limited anthropogenic impact did not carry any ESBL‐producing Enterobacteriales (Kozoderović et al., 2021). Thus, frequencies of resistant Enterobacteriales among raptors appear to reflect frequencies, where available, in their geographical area and level of anthropogenic impact.

Forty‐six of the ESBL *E. coli* isolates harbored the *bla*_CTX‐M‐15_ gene (Figure 2a). Two other isolates harbored *bla*_CTX‐M‐27_, one isolate *bla*_CTX‐M‐55_ and one isolate *bla*_CTX‐M‐101_ (Figure 2a). The high prevalence of the ESBL gene *bla*_CTX‐M‐15_ isolated from the vultures in this study is in line with the results in a recent systematic review assessing the epidemiology of ESBL‐ and carbapenem‐producing Enterobacteriales among humans, animals, and the environment in West and Central Africa that concluded that the *bla*_CTX‐M‐15_ was the predominant gene (Ouchar Mahamat et al., 2021). Only two of the *E. coli* isolates (3009 and 3082) did not harbor any *bla*_CTX‐M_ genes, both belonging to sequence type (ST)−656 and instead harbored *bla*_SHV‐11_ genes (Figure 2a). Among the 52 *E. coli* isolates with extended‐spectrum cephalosporin resistance, genes for plasmid‐mediated quinolone resistance (*qepA4* or *qnrS1*) were detected in 47 (90.4%), whereas phenotypic resistance toward ciprofloxacin was detected in 65.4% (*n* = 34) (Figure 2a, Supporting Information: Table S1 at https://doi.org/10.5281/zenodo.7645125). No resistance genes conveying resistance toward colistin were found.

### 3.2 Genotypic clustering using MLST, cgMLST, and phylogroup analysis

**Figure 3:**
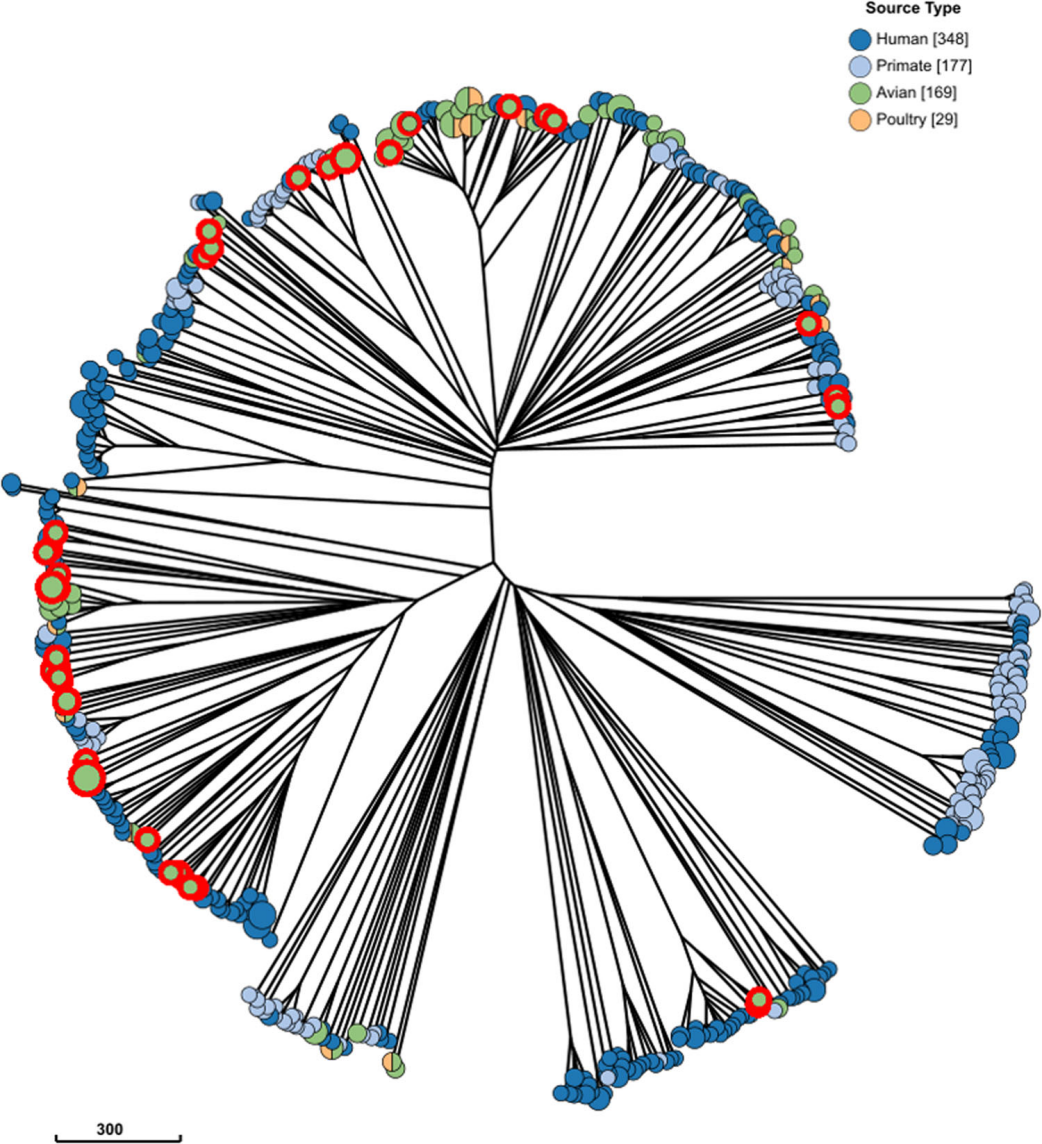
The minimum spanning tree of hierarchical clustering of cgMLST analysis of 723 *Escherichia coli* isolates in Enterobase isolated from human (dark blue), primate (light blue), avian (green), and poultry (orange) in Gambia. Red circles indicate ESBL *E. coli* isolates collected from hooded vultures in this study.

In total, the 52 sequenced *E. coli* isolates belonged to 23 different MLSTs, with clustering in ST‐196 and ST‐540 for 12% (*n* = 6), respectively, ST‐202 for 14% (*n* = 7) and ST‐746 for 17% (*n* = 9) of the isolates. Sequence types of clinical significance known to cause human infections, such as ST‐10, commonly associated with mcr‐1 carriage (Matamoros et al., 2017), and ST‐38, frequently associated with *bla*_OXA‐48_ carriage (Turton et al., 2016) were found among the hooded vultures. All isolates belonging to the same ST also had the same phylogroup (Figure 2a). Performing hierarchical clustering of cgMLST data, including all *E. coli* isolates from the Gambia in Enterobase (*n* = 723) collected from human, primate, and avian sources, shows a widespread distribution of the isolates from this study (Figure 3). The hooded vultures in this study thus carry ESBL‐producing *E. coli* representing a large fraction of the currently known genetic diversity of the existing *E. coli* population in the Gambia, indicating inter‐species exchange.

### 3.3 Plasmid replicons, mobile genetic elements, and genetic context

**Figure 4:**
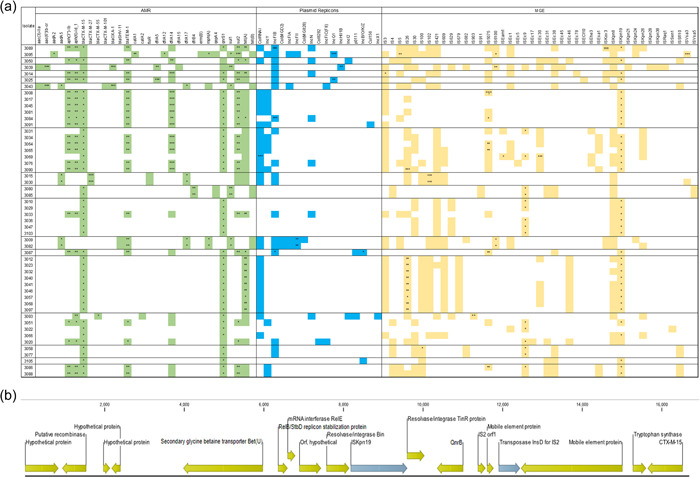
Genetic context of antimicrobial resistance (AMR), plasmid replicons, and mobile genetic elements for each isolate (a) and a schematic illustration of the genetic context surrounding the *bla*_CTX‐M‐15_ gene (b).

Several different plasmid replicons were identified among the *E. coli* isolates (Figure 4a). For a few isolates, the genetic context could be identified, but in most cases, not. In isolate 3087, plasmid replicons IncFIB and IncB/O/K/Z were identified in the same genetic context (i.e., same contig) as *bla*_*CTX‐M‐15*,_ *qnrS1*, *tet(A)*, and IS*Kpn19* (Figure 4a,b).

For the two *E. coli* isolates with *bla*_CTX‐M‐27_, the gene was located on the same contig as IS102 (Figure 4a). A recent systematic review of resistome epidemiology in Africa reports that MGEs in association with ARGs were rarely described in isolates from environmental and animal samples (Sekyere & Reta, 2020). In this study, 84.7% (*n* = 39/46) of isolates with *bla*_CTX‐M‐15_, the gene was colocalized with *qnrS1* and IS*Kpn19* (Figure 4a,b). This genetic context has been described by others, including an IncFII *bla*_CTX‐M‐15_‐harboring plasmid identified from *Shigella sonnei* in Switzerland (Campos‐Madueno et al., 2020). Another study has identified the spread of a novel plasmid containing *bla*_CTX‐M‐15_ together with *bla*_TEM‐1_ and *qnrS1* (Fortini et al., 2015).

**Figure 5:**
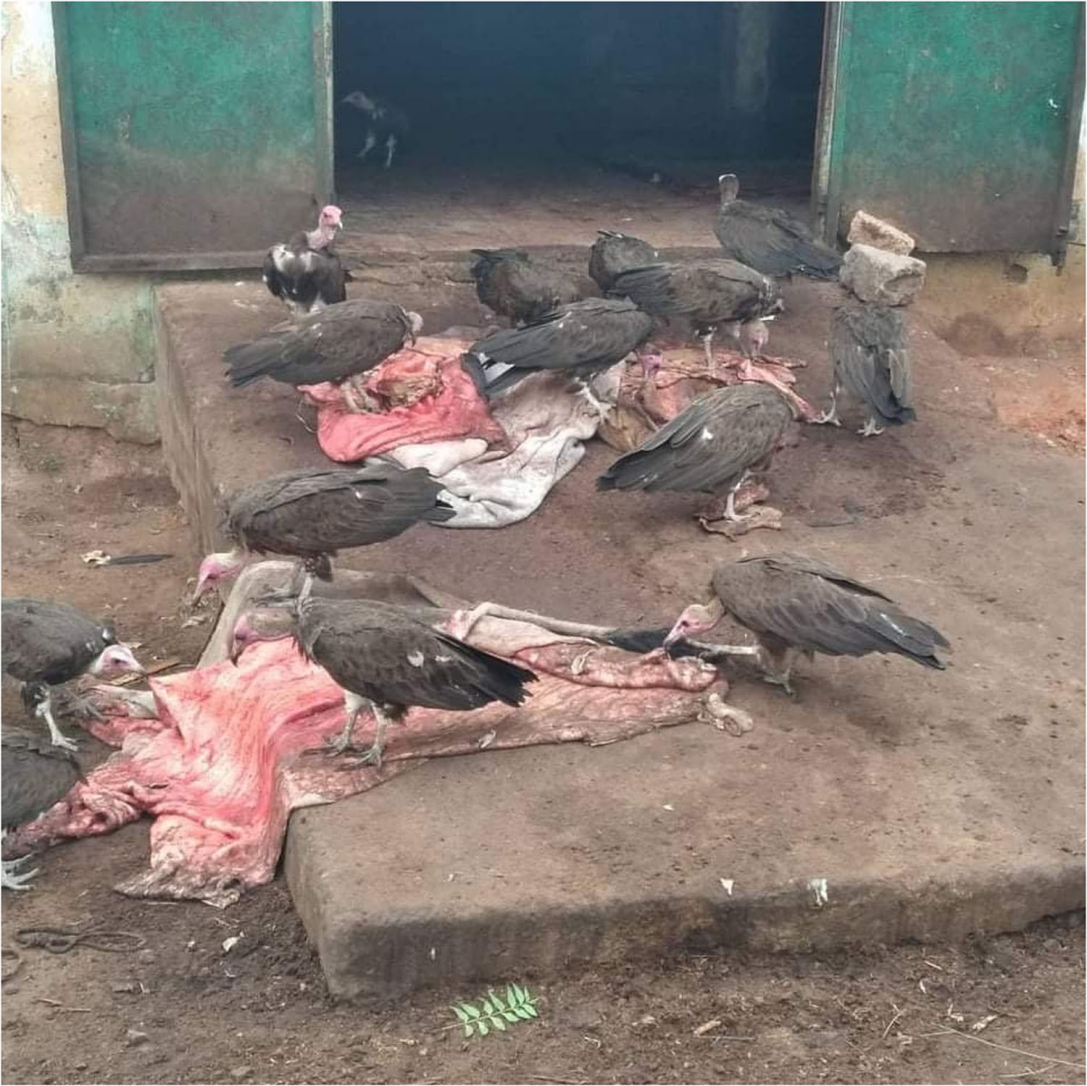
Hooded vultures scavenging nearby and in slaughterhouses show anthropogenic interaction posing a risk for further dissemination in the environment and further transmission to humans and animals (photograph taken by F. Camara).

The presence of ARB in wild animals is affected by different biological, ecological, and geographic factors, still warranting further investigations to fully understand dissemination patterns and selection pressure (Arnold et al., 2016; Lee et al., 2020; Mughini‐Gras et al., 2019; Wang et al., 2017). Global monitoring through metagenomic analysis of urban sewage revealed a correlation between total AMR abundance and socioeconomic factors, where the highest levels of AMR were found in countries from the African continent (Hendriksen et al., 2019). This has also been shown in a cross‐country regression analysis where lower AMR abundance correlated with better infrastructure, access to clean water, and improved sanitation (Laxminarayan et al., 2020). The data in this study implicate that vultures could act as bioindicators for circulating ARB and ARG in anthropogenic‐affected environments. Wild animals living and feeding in human‐affected environments are often colonized with ARB of great clinical significance (Dolejska & Literak, 2019; Karesh et al., 2012). Aquatic environments and wastewater treatment plants are often highlighted for the risk of AMR transmission between humans and the environment (Bengtsson‐Palme et al., 2019; Calero‐Cáceres et al., 2022). This study implies that nonaquatic species such as scavengers and opportunistic feeders in close proximity to anthropogenic environments could pose a risk of AMR transmission between animals, the environment, and humans. In the Gambia, Banjul area, the hooded vultures, apart from feeding from landfills and human waste, are often observed to scavenge close to slaughterhouses and food markets (Figure 5). In addition to also having nightly roosting trees in the middle of human settlements, hooded vultures are not only bioindicators for AMR but could also be potential vectors for AMR transmission within different one‐health sectors. On the other hand, hooded vultures, as scavengers, remove carcasses that potentially carry pathogenic microorganisms, thereby possibly reducing such a threat to human and animal health. Furthermore, both avian and human pathogenic *E. coli* strains share a lot of common virulence factors (Kathayat et al., 2021). An interspecies exchange of *E. coli* strains is, of course, also concerning from a conservation perspective since it might include virulent *E. coli* strains that could be devastating for a critically endangered species such as the hooded vulture. A recent review investigated the impact of microorganisms on vultures and concluded that their health could be affected by both human pathogenic bacteria and viruses, possibly influencing fitness and mortality (Plaza et al., 2020). Specifically, hooded vultures infected by avian influenza (H5N1) had neurological symptoms, and white‐rumped vultures showed signs of enteritis caused by *E. coli* (Plaza et al., 2020).

## 4 CONCLUSION

This study has identified a high prevalence of ESBL‐producing *E. coli* among hooded vultures in the Gambia. The isolates identified were distributed over a large fraction of the currently known genetic diversity of the existing *E. coli* population in the Gambia collected from human, primate, and avian sources. However, there was a low diversity regarding ESBL genes with *bla*_CTX‐M‐15_ colocalized with IS*Kpn19* dominating across different STs, and no carbapenemases were found. Based on data from this report, hooded vultures, as scavengers and top predators, may function as bioindicators for the presence of ARB in relation to anthropogenic and modified environments. Furthermore, this study highlights how exposed vultures are to anthropogenic impact, not only posing a threat to the birds but also as they may serve as vectors for further dissemination of ARB in the environment and (re‐)transmission of ARB to humans. Future research should focus on possible mitigation strategies to reduce the risk of AMR dissemination by a hooded vulture, with special attention on how to minimize bird interaction with potential point sources of AMR and areas where the risk of AMR transmission between birds and humans is high. The conservation aspect of hooded vultures must be given particular attention when forming mitigation strategies for AMR transmission between different One Health sectors since the vultures are not only at risk of being vectors for AMR transmission but also pose a risk of being exposed to harmful zoonotic pathogens.

## AUTHOR CONTRIBUTIONS

**Hanna Woksepp**: Conceptualization (supporting); data curation (lead); formal analysis (equal); investigation (equal); methodology (equal); project administration (equal); resources (equal); software (equal); supervision (equal); validation (equal); visualization (lead); writing—original draft (lead); writing—review and editing (equal). **Fagimba Camara**: Conceptualization (equal); formal analysis (equal); funding acquisition (supporting); project administration (supporting); resources (equal); writing—review and editing (equal). **Jonas Bonnedahl**: Conceptualization (lead); data curation (supporting); formal analysis (equal); funding acquisition (lead); investigation (equal); methodology (equal); project administration (equal); resources (lead); software (equal); supervision (equal); validation (equal); visualization (equal); writing—original draft (equal); writing—review and editing (equal).

## CONFLICT OF INTEREST STATEMENT

The authors declare no conflict of interest.

## ETHICS STATEMENT

An export permit for feces from hooded vultures was issued by Wildlife Conservation Department, the Gambia, West Africa Regulation Overleaf DPWM. No 000401 to F. Camara 14th Nov 2019. An import permit for J. Bonnedahl was issued by the Swedish Board of Agriculture, DNR 6.7.18‐14663/2019.

## Data Availability

All data are provided in full in this paper except for the data in Supporting Information: Table S1 (Metadata and antimicrobial data), which are available in the Zenodo repository at https://doi.org/10.5281/zenodo.7645125, and the sequences available in the European Nucleotide Archive (ENA) under accession number PRJEB52194: https://www.ebi.ac.uk/ena/browser/view/PRJEB52194
